# Silencing of Oleuropein β-Glucosidase Abolishes the Biosynthetic Capacity of Secoiridoids in Olives

**DOI:** 10.3389/fpls.2021.671487

**Published:** 2021-09-03

**Authors:** Konstantinos Koudounas, Margarita Thomopoulou, Aimilia Rigakou, Elisavet Angeli, Eleni Melliou, Prokopios Magiatis, Polydefkis Hatzopoulos

**Affiliations:** ^1^Laboratory of Molecular Biology, Department of Biotechnology, Agricultural University of Athens, Athens, Greece; ^2^Laboratory of Pharmacognosy and Natural Products Chemistry, Department of Pharmacy, National and Kapodistrian University of Athens, Athens, Greece

**Keywords:** *Olea europaea*, olive, secoiridoids, oleuropein, β-glucosidase, virus-induced gene silencing, tobacco rattle virus, one-dimensional quantitative nuclear magnetic resonance (1D-qNMR)

## Abstract

Specialized metabolism is an evolutionary answer that fortifies plants against a wide spectrum of (a) biotic challenges. A plethora of diversified compounds can be found in the plant kingdom and often constitute the basis of human pharmacopeia. Olive trees (*Olea europaea*) produce an unusual type of secoiridoids known as oleosides with promising pharmaceutical activities. Here, we transiently silenced oleuropein β-glucosidase (OeGLU), an enzyme engaged in the biosynthetic pathway of secoiridoids in the olive trees. Reduction of *OeGLU* transcripts resulted in the absence of both upstream and downstream secoiridoids *in planta*, revealing a regulatory loop mechanism that bypasses the flux of precursor compounds toward the branch of secoiridoid biosynthesis. Our findings highlight that *OeGLU* could serve as a molecular target to regulate the bioactive secoiridoids in olive oils.

## Introduction

Olive (*Olea europaea* L., Oleaceae) is an emblematic crop of the Mediterranean Basin and over the last decades, the cultivation has expanded to the Americas, Asia, and Oceania mostly due to the high nutritional value of olive oil. An essential trait that evolution has gifted to olives is the production of a certain type of secondary metabolites, known as oleosides, with an extremely narrow taxonomic distribution. Oleosides are terpene-derived secoiridoids, conjugates of glycosylated elenolic acid with a characteristic exocyclic 8,9-olefinic bond (Soler-Rivas et al., 2000), and are only present in the Oleaceae family and the genus *Caiophora* (Loasaceae) (Obied et al., 2008). Oleoside derivatives synergistically contribute to the beneficial aspects of olive oil in human health (Tripoli et al., 2005; Papanikolaou et al., 2019) and determine the flavor and quality of olive oil (Vitaglione et al., 2015). Recently, the European Medicinal Agency published a risk-benefit report on olives, and the European Food Safety Authority has already approved a health claim related to polyphenols in olive oil (EFSA, 2012; HMPC, 2017).

The dominant secoiridoid in olives accumulating up to 14% in young drupes is oleuropein (Amiot et al., 1986), an ester of elenolic acid with 2′-(3′,4′-dihydroxyphenyl)ethanol (hydroxytyrosol) that exhibits antioxidant, anti-inflammatory, antiproliferative, antimicrobial, and antiviral activities, hence being a metabolite of high interest for humans (Bulotta et al., 2013). The first identified enzyme that is engaged in the metabolism of oleuropein is the oleuropein-specific β-glucosidase (OeGLU; E.C. 3.2.1.206) (Koudounas et al., 2015, 2017). OeGLU is a homomultimeric enzyme, member of the Glycoside Hydrolase 1 family (GH1), and localized in the nucleus. Oleuropein is localized in the vacuoles or cytosol (Konno et al., 1999), thus physically separated from the OeGLU enzyme, and upon cell disruption, the dual-partner defensive system comes in contact. Deglycosylation of oleuropein by OeGLU produces an unstable aglycone form that is rapidly converted to a highly reactive molecule with a glutaraldehyde-like structure. This compound covalently binds to amino acids and exhibits strong protein denaturing/cross-linking activities, providing a mighty arsenal against herbivores (Konno et al., 1999).

In addition to a pivotal role in the plant chemical defense of Oleaceae species, the OeGLU-mediated enzymatic detonation of oleuropein is also crucial during olive oil extraction. Crushing of olive drupes followed by malaxation (coalescence of oil droplets through the mixing of olive paste) causes cell disruption. Oleuropein is exposed to OeGLU, and the aglycone products are massively produced, thus shaping the organoleptic properties of olive oil (Obied et al., 2008; Romero-Segura et al., 2012; Hachicha Hbaieb et al., 2015; Vitaglione et al., 2015).

**FIGURE 1:**
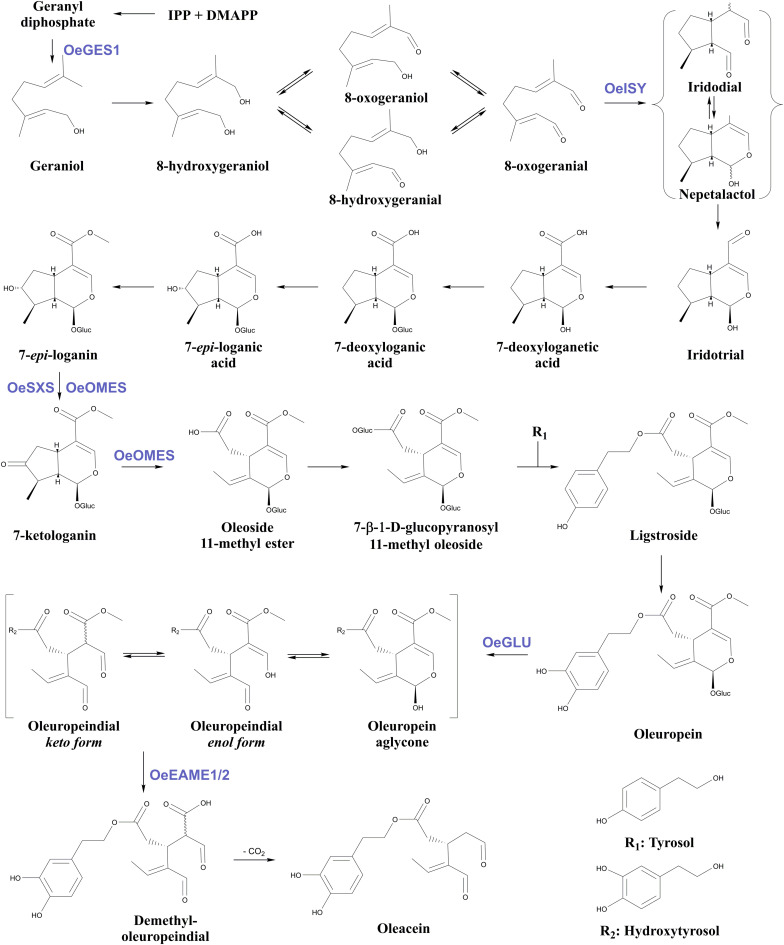
Proposed biosynthetic pathway of oleuropein and oleacein in Oleaceae. Characterized enzymes of the pathway as referred in the literature (Obied et al., 2008; Vezzaro et al., 2012; Koudounas et al., 2015; Alagna et al., 2016; Volk et al., 2019; Rodríguez-López et al., 2021). IPP, isopentenyl diphosphate; DMAPP, Dimethylallyl diphosphate; Glu, glucose; OeGES1, geraniol synthase; OeISY, iridoid synthase; OeOMES, oleoside methyl ester synthase; OeSXS, secoxyloganin synthase; OeGLU, oleuropein β-glucosidase; OeEAME1/2, elenolic acid methylesterase 1 and 2.

Apart from OeGLU, only three enzymatic hubs are known to be engaged in the biosynthesis and biotransformations of secoiridoids in olive. The first one is an iridoid synthase (OeISY) which converts 8-oxogeranial into the iridoid scaffold in a two-step reduction-cyclization sequence (Alagna et al., 2016). Recently, two bi-functional cytochrome P450s (oleoside methyl ester synthase, OeOMES and secoxyloganin synthase, OeSXS) which convert 7-*epi*-loganin into the secoiridoid scaffold in two sequential oxidation steps were characterized (Rodríguez-López et al., 2021). Additionally, two methylesterase enzymes (elenolic acid methylesterase 1, OeEAME1 and elenolic acid methylesterase 2, OeEAME2) that generate oleacein and oleocanthal after the concerted activity of β-glucosidase on oleuropein and ligstroside, respectively, have been identified (Volk et al., 2019). Finally, a *bona fide* geraniol synthase (OeGES1) has been reported to generate geraniol – monoterpene alcohol that is the precursor of iridoids (Vezzaro et al., 2012). Despite the recent advances toward the elucidation of this pathway, the proposed biosynthetic pathway of oleuropein in Oleaceae involves at least 18 enzymatic steps, therefore, remains largely uncharacterized (Figure 1).

The olive tree is a non-model plant, member of a plant family that comprises perennial woody species (Green, 2004) recalcitrant to genetic transformation. Therefore, studies on the Oleaceae-specific biosynthetic pathway of secoiridoids and characterization of the aforementioned enzymatic hubs have been limited to heterologous *in vivo* systems (i.e., *Nicotiana benthamiana*, *Saccharomyces cerevisiae*, and *Escherichia coli*) and *in vitro* enzymatic assays, thus raising the question of the actual contribution of these enzymes in olives. Plant genomes are known to typically encode more than 30 members of the GH1 family (Ketudat Cairns and Esen, 2010) therefore olive possess other β-glucosidases that could deglycosylate oleuropein. However, we have previously determined that *OeGLU* is a single copy gene in the olive genome (Koudounas et al., 2015), and defensive GH1 β-glucosidases are known to be highly diversified and exhibit exceptional specificity against their respective substrates (Verdoucq et al., 2004; Xia et al., 2012).

In this study, we recruited the recently described *Agrobacterium*-mediated virus-induced gene silencing (VIGS) methodology (Koudounas et al., 2020) to transiently silence *OeGLU* in olive seedlings and validate whether OeGLU is the major oleuropein β-glucosidase *in planta*. Besides, downregulation of the OeGLU enzyme, transient silencing of OeGLU unexpectedly affected the biosynthesis of upstream and downstream secoiridoids suggesting the existence of a feedback regulatory loop in this pathway.

## Materials and Methods

### Plant Material and Growth Conditions

Ripe fruits were harvested from *Olea europaea* L. cv. “Koroneiki” grown in a natural environment at the Agricultural University of Athens. The black mesocarps were removed, and the woody endocarps were subjected to 10% sodium hydroxide for 10 min and then thoroughly washed to remove any fleshy remnants. The endocarps were dried naturally and stored until use. Seeds were extracted after gently cracking the woody endocarp with a bench vice. After surface sterilization followed by stratification for 7 days at 4°C, seeds were transferred to soil for germination and grown at 22°C in a Fitotron growth chamber (Weiss Gallenkamp, Loughborough, United Kingdom) with a 16/8 h light/darkness cycle and 100 μmol m^–2^ s^–1^ light intensity.

### Plasmid Construction

The pTRV1 (stock no.: CD3-1039) and pTRV2-MCS (stock no.: CD3-1040) plasmids encoding the bipartite RNA genome of tobacco rattle virus (TRV) were obtained from the Arabidopsis Biological Resource Center (ABRC^1^). RNA was extracted from olive young leaves using a phenol/chloroform procedure, and fragments of *OeGLU* (211 bp) and *OeChlH* (297 bp) were amplified by real-time polymerase chain reaction (RT-PCR) using the OeGLUi-F and OeGLUi-R, or OeChlH-F and OeChlH-R primers, respectively (Supplementary Table 1). To produce the pTRV2-OeGLU and pTRV2-OeChlH constructs, the PCR products were blunt-end ligated into the *Sma*I site of pUC19, and after verification by sequencing, the PCR products were directly sub-cloned into pTRV2-MCS (empty vector, EV) in antisense orientation relative to the TRV coat protein, utilizing the *Kpn*I and *Xba*I restriction sites of pUC19.

### Agrobacterium-Mediated VIGS

The pTRV1 and pTRV2 constructs with either fragment of the targeted genes for silencing or an EV were electroporated in the *Agrobacterium tumefaciens* strain C58C1 Rif^*R*^ (GV3101) containing the T-DNA-deficient Ti plasmid pMP90. Positive transformants were harvested, and cells harboring the pTRV1 plasmid were mixed 1:1 with cells harboring the pTRV2 constructs in inoculation medium (10 mM 2-N-morpholino-ethanesulfonic acid pH 5.6, 10 mM MgCl_2_, and 150 μM acetosyringone). Olive plantlets having at least one pair of fully expanded leaves were Agroinoculated by gently pricking the leaves at the abaxial side as described (Koudounas et al., 2020). This procedure was repeated every 2 weeks until the observation of a phenotype. Leaves from the first two leaf pairs that emerged after Agroinoculation were harvested and used for molecular and biochemical studies. The presence of TRV in non-Agroinoculated (i.e., newly emerged) leaves was validated by detecting the transcripts of TRV coat protein (Supplementary Table 1; Senthil-Kumar and Mysore, 2014).

### Gene Expression Analysis

Reverse transcription was performed with SuperScript II RT (Invitrogen, Life Technologies, Carlsbad, CA, United States) using 200 ng of DNA-free RNA and the oligo(dT)_17_ primer. Quantitative gene expression analysis was performed in a PikoReal 96 Real-Time PCR system (Thermo Fisher Scientific, Waltham, MA, United States) using the SYBR Select Master Mix (Applied Biosystems, Life Technologies, Carlsbad, CA, United States) and calculated by the ΔΔCt method. *OeGLU*, *OeGES1*, *OeISY*, *OeOMES*, *OeSXS*, and *OeEAME1/2* transcripts were amplified with the OeGLUq-F and OeGLUq-R, OeGES1q-F and OeGES1q-R, OeISYq-F and OeISYq-R, OeOMESq-F and OeOMESq-R, OeSXSq-F and OeSXSq-R, OeEAME1q-F and OeEAME1q-R, or OeEAME2q-F and OeEAME2q-R primers, respectively (Supplementary Table 1). Normalization of gene expression data was performed by using the *OeActin* housekeeping gene as a reference with primers OeActin_q-F and OeActin_q-R (Supplementary Table 1). Standard curves for both the target and the reference genes were generated to determine the amplification efficiency of each gene.

### Enzymatic Assays

Harvested leaves were frozen in liquid nitrogen and ground into powder using a mortar and pestle. Crude proteins were extracted in ice-cold extraction buffer (100 mM Tris–HCl, pH 8.8, 100 mM EDTA, 1 mM PMSF, 100 mM KCL, 10 mM Na_2_SO_3_, and 100 mM glycine), samples were centrifuged, and the soluble fractions were quantified by the Bradford assay. The enzymatic reactions with oleuropein were performed by incubating 10 μg of the soluble protein extracts in 100-μl hydrolysis buffer (5 mM oleuropein, 150 mM sodium acetate, pH 5.5, and 0.05% bovine serum albumin [BSA]) at 37°C for 6 min. The deglycosylation degree of oleuropein was measured by high-performance liquid chromatography (HPLC) as described previously (Koudounas et al., 2015). The enzymatic reactions with *p*-nitrophenyl β-D-glucopyranoside (pNPGlu) were performed by incubating 10 μg of the soluble protein extracts in 500 μl hydrolysis buffer (10 mM pNPGlu, 150 mM sodium acetate, pH 5.5, and 0.05% BSA) at 37°C for 30 min, and reactions were stopped by mixing with an equal volume of 0.2 M sodium carbonate. The deglycosylated *p*-nitrophenyl was determined by measuring the absorbance at 405 nm. Relative activity was calculated by arbitrarily setting the oleuropein or pNPGlu β-glucosidase activity of soluble extracts from plants Agroinoculated with pTRV2-EV to 100%.

### Chemicals and Standards

All solvents were of analytical grade and purchased from Merck. Syringaldehyde (98% purity) used as internal standard (IS) was purchased from Sigma-Aldrich (Steinheim, Germany). IS solution was prepared in acetonitrile at a concentration of 0.5 mg/mL and kept in a refrigerator. Prior to use, the IS solution was left to come to room temperature. Oleuropein was purchased from Extrasynthese (Genay, France), stock solution (15 mM) was prepared in ddH_2_O, and aliquots were stored in −20°C until usage.

### Extraction and NMR Spectra Analysis

Olive leaves were dried at room temperature in a dark place for a week. Then, the leaves were pulverized at room temperature, and 20 ml of MeOH were added to 100 mg of powdered dried olive leaves. The mixture was placed for 45 min in an ultrasonic bath followed by centrifugation at 4,000 rpm for 3 min. A portion of the methanolic extract was collected (10 mL), and 0.5 ml syringaldehyde in acetonitrile solution was added. The mixture was evaporated to dryness under vacuum in a rotary evaporator. The residue of the above procedure was dissolved in MeOD (600 μl), transferred to a 5 mm NMR tube, and the ^1^H NMR was recorded at 400 MHz. Oleuropein was identified and quantitated by integrating the peak of proton at 5.9 ppm, and based on the equation y = 0.512x + 0.0904, the results were expressed per 100 mg olive leaf sample (Mousouri et al., 2014). NMR spectra were recorded on a DRX 400 MHz and analyzed with MestreNova. A total of 32 scans were collected into 32K data points over a spectral width of 0–16 ppm with a relaxation delay of 1 s and an acquisition time of 1.7 s. Prior to Fourier transformation (FT), an exponential weighting factor corresponding to a line broadening of 0.3 Hz was applied. The spectra were phase corrected, and accurate integration was performed manually for the peaks of interest.

### Statistical Analysis

Three technical replicates from three biological replicates per treatment were used in real-time quantitative polymer chain reaction (RT-qPCR) analyses and *in vitro* enzymatic assays. Three or six biological replicates of plants Agroinoculated with pTRV2-EV or pTRV2-OeGLU, respectively, were used for NMR spectra analysis. Data were analyzed using the GraphPad Prism program (GraphPad Software). The statistical significance of differences between control and *OeGLU*-silenced samples was tested by unpaired Student’s *t*-test.

### Accession Numbers

Sequence data discussed in this article are available in GenBank under accession numbers: AY083162 (OeGLU), GABQ01080755 (OeChlH), GABQ01079399 (OeActin), AF406990 (pTRV1), AF406991 (pTRV2-MCS), JN408072 (OeGES1), KT954038 (OeISY), MT909123 (OeOMES), MT909125 (OeSXS), MK234850 (OeEAME1), and MK160486 (OeEAME2).

## Results

### TRV-Mediated Silencing of *OeGLU*

To silence *OeGLU*, we first performed bioinformatic analysis to identify specific regions that would not trigger any off-target silencing (Supplementary Figure 1). cDNA fragments of either *OeGLU* or the H subunit of Mg-protoporphyrin chelatase (*OeChlH*) were cloned in plasmids encoding the bipartite RNA genome of TRV. ChlH is involved in the biosynthesis of chlorophyll and serves as a positive control of the successful VIGS process (Senthil-Kumar and Mysore, 2014). Since the fragment used to trigger silencing of *OeChlH* had significant homology (Supplementary Figure 2A) with the corresponding cDNA from Nicotiana benthamiana (*NbChlH*), the efficiency of the construct to trigger silencing of *ChlH* was firstly validated in Agroinfiltrated tobacco plants. As expected, a yellowish leaf phenotype due to chlorophyll reduction was observed (Supplementary Figure 2B) indicating that *NbChlH* was successfully silenced. We next validated the efficiency of the TRV-based VIGS constructs by Agroinoculating olive seedlings. An intense yellowing phenotype indicative of successful silencing of the targeted gene in olive plants Agroinoculated with the *pTRV2-OeChlH* construct confirmed the successful application of the methodology (Supplementary Figure 3). Olive plants Agroinoculated with either the pTRV2-EV or the pTRV2-OeGLU constructs had no detectable phenotype (Supplementary Figure 3). Detection of the TRV coat protein transcripts in non-Agroinoculated leaves confirmed that TRV successfully infected the olive plants and moved systemically toward newly emerged leaves (Supplementary Figure 4).

**FIGURE 2:**
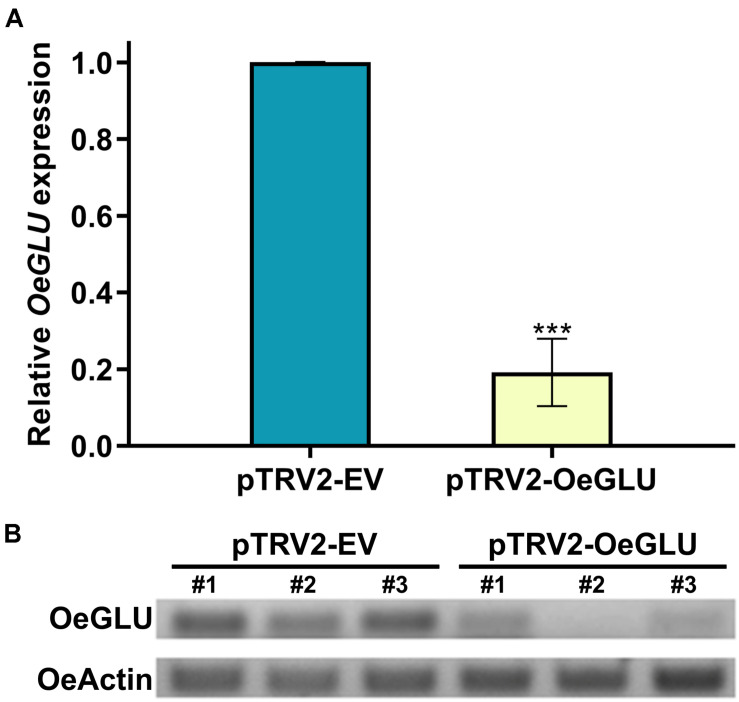
VIGS-mediated silencing of *OeGLU* in olive plants. Real Time quantitative PCR (RT-PCR) **(A)** and semi-quantitative RT-PCR **(B)** analysis of *OeGLU* expression level of plants Agroinoculated with either pTRV2-EV or pTRV2-OeGLU constructs. OeActin was used as an internal control to normalize the samples. Three technical replicates of three biological replicates per treatment **(A)** or three biological replicates per treatment **(B)** were analyzed. Mean ± SE and asterisks denote statistical significance (***P ≤ 0.001; Student’s *t*-test). Numbers indicate biological replicates. EV, empty vector; OeGLU, oleuropein β-glucosidase.

To analyze the degree of silencing of *OeGLU*, we performed RT-qPCR analysis in olive young leaves, which clearly demonstrated that *OeGLU* was successfully silenced and the expression level was reduced by 80.82% compared to control conditions (Figure 2A). This result was further validated by semi-quantitative RT-PCR (Figure 2B). Additionally, we performed RT-qPCR analysis to monitor the expression levels of other characterized enzymes from the secoiridoid pathway in these plants. Silencing of *OeGLU* did not affect the transcript levels of either upstream (i.e., OeGES1, OeISY, and OeOMES) or downstream (i.e., OeEAME2) enzymes (Supplementary Figure 5).

### Silencing of *OeGLU* Drastically Affects the Degree of Oleuropein Deglycosylation *in vitro*

**FIGURE 3:**
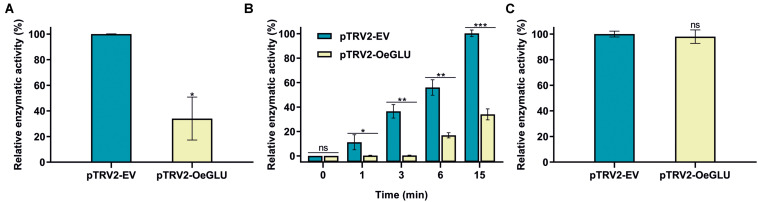
*In vitro* enzymatic assays of crude leaf protein extracts of olives Agroinoculated with either pTRV2-EV or pTRV2-OeGLU constructs. **(A)** Relative enzymatic activity against oleuropein at 6 min. **(B)** Relative enzymatic activity against oleuropein at regular time intervals. **(C)** Relative enzymatic activity against pNPGlu. Three technical replicates of three biological replicates per treatment were analyzed. Mean ± SE and asterisks denote statistical significance (***P ≤ 0.001, **P ≤ 0.01, *P ≤ 0.05, ns: not significant; Student’s *t*-test). EV, empty vector; OeGLU, oleuropein β-glucosidase; pNPGlu, *p*-nitrophenyl β-D-glucopyranoside.

We next investigated whether this biochemical phenotype would have an impact on the deglycosylation of oleuropein. HPLC analysis of the *in vitro* enzymatic assays using protein extracts from plants that *OeGLU* was silenced with oleuropein demonstrated that the relative *OeGLU* enzymatic activity was drastically reduced (Figure 3A). The degree of deglycosylation of oleuropein was reduced by 65.96% compared to control conditions after 6 min of incubation. As expected, this result coincides with the RT-qPCR (Figure 2A) and is in accordance with the abundance of *OeGLU* transcripts in the RNAi-silenced plants. The progress of the enzymatic deglycosylation of oleuropein revealed that the relative activity of OeGLU during the first 3 min of incubation was null, at 6 min was 30.09%, and even after 15 min of incubation, it remained at 33.93% compared to control (Figure 3B).

This result can be attributed to the fact that the VIGS-based approach of silencing genes varies and rarely reaches 100% effectiveness, as would be in a knockout mutant. Therefore, a number of remaining “escaping” transcripts were translated to the reduced quantity of OeGLU, nevertheless adequate to catalyze the enzymatic reaction to less extend. In addition, other non-specific β-glucosidases present in the olive tree that exhibit a lower affinity for oleuropein could potentially deglycosylate this substrate but within a worth noting delayed time lapse.

The specificity of silencing of *OeGLU* was validated by screening for the relative enzymatic activity of pNPGlu deglycosylation – a general synthetic substrate often used with β-glucosidases (Figure 3C). Since the relative non-specific β-glucosidase activity of the crude extracts remained at similar levels, the silencing of *OeGLU* was highly selective.

### Silencing of *OeGLU* Abolishes the Biosynthesis of Both Upstream and Downstream Secoiridoids *in planta*

**FIGURE 4:**
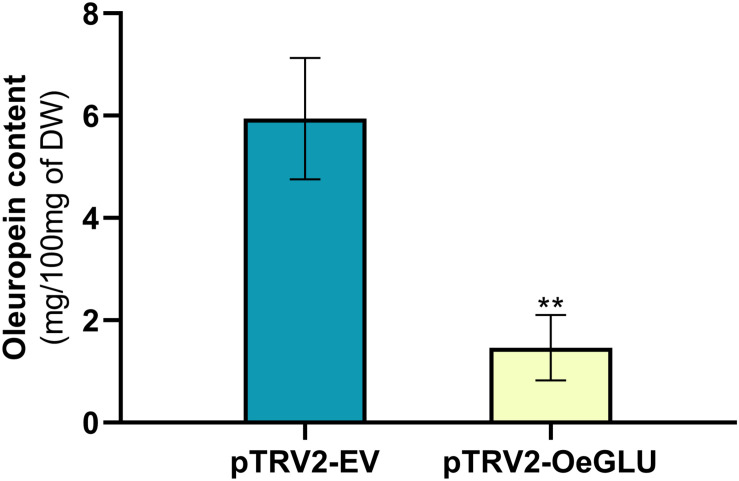
Oleuropein content (mg) per 100 mg (dry weight) of olive leaves. Olive plantlets Agroinoculated either with pTRV2-EV (*n* = 3) or with pTRV2-OeGLU (*n* = 6) constructs were screened (Supplementary Table 2). Mean ± SE and asterisks denote statistical significance (**P ≤ 0.01; Student’s *t*-test). EV, empty vector; OeGLU, oleuropein β-glucosidase.

**FIGURE 5:**
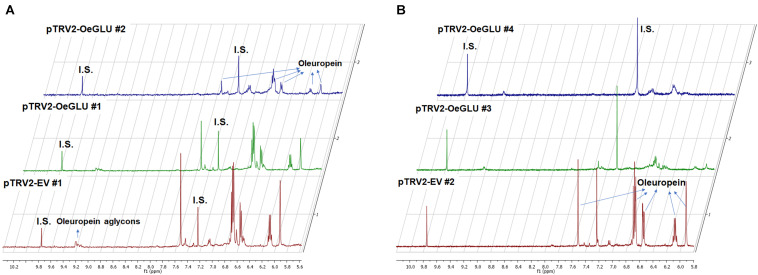
Representative 1D-qNMR spectra of the *in planta* oleuropein content. Comparison between the methanolic extracts of olive plants Agroinoculated with pTRV2-EV or pTRV2-OeGLU constructs **(A,B)**. Numbers indicate biological replicates. EV, empty vector; IS, internal standard; OeGLU, oleuropein β-glucosidase; 1D qNMR, one-dimensional quantitative nuclear magnetic resonance.

We next questioned whether the reduction of OeGLU would have an impact *in planta*. Instead of substrate accumulation, the content of the oleuropein secoiridoid in *OeGLU* silenced plants was lower compared to control non-silenced plants (Figure 4 and Supplementary Table 2). In certain plants, oleuropein was even non-detectable as observed in the NMR spectra of the leaf extracts (Figures 4, 5A,B; Supplementary Figure 6). In our experimental setup, the lowest concentration of oleuropein that can be quantified is 70 μg of oleuropein per 100 mg of dry weight (Mousouri et al., 2014), therefore in these plants the amount of oleuropein was reduced by at least 1,000 times compared to control plants. Additionally, the content of oleuropein aglycones and any related downstream secoiridoid were almost non-detectable. This result is in agreement with the low amount of the precursor oleuropein since the concerted activity of OeGLU on oleuropein followed by methylesterase activity results in a pool of aglycone derivatives (Supplementary Figure 7). It is worth noting that the existence of the oleuropein aglycones *in planta* (Figure 5A) has not been highlighted in the literature potentially pointing out a continuous, nevertheless at a low level, catabolic pathway of oleuropein. How is oleuropein transported from the vacuoles to the nucleus to be deglycosylated by OeGLU and especially how the cells cope with the highly reactive aglycones remain to be addressed.

**FIGURE 6:**
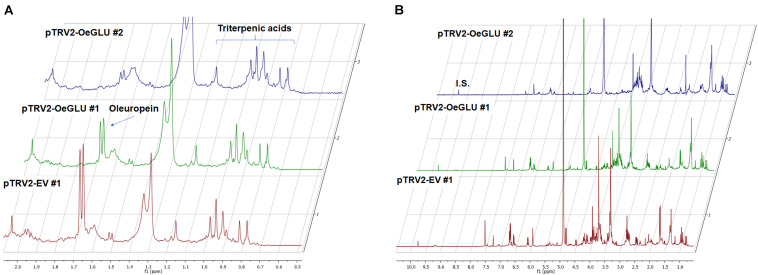
Representative 1D-qNMR spectra. Comparison between the methanolic extracts of plants Agroinoculated with pTRV2-EV and pTRV2-OeGLU constructs for their triterpenic acids content **(A)** or their full spectrum **(B)**. Numbers indicate biological replicates. EV, empty vector; IS, internal standard; OeGLU, oleuropein β-glucosidase; 1D-qNMR, one-dimensional quantitative nuclear magnetic resonance.

In contrast, oleanolic acid or maslinic acid (triterpenic acids with characteristic peaks observed between 0.8 and 1.2 ppm), secondary compounds not related to the biosynthetic pathway of secoiridoids, were unaffected by the silencing of *OeGLU* (Figure 6A). The methyl group of oleuropein observed at 1.7 ppm and the characteristic H-1 at 5.90 ppm presented significant quantitative differences among the silenced and the control samples confirming that the effect *in planta* was apparently secoiridoid specific. No other peaks of related secoiridoids of any precursor molecule could be observed in the plants that *OeGLU* was silenced (Figures 5A,B, 6B). Control Agroinoculated plants contained high amount of oleuropein, and a detectable amount of decarboxymethylated oleuropein aglycone products, which are observed in the aldehyde region (Figures 4, 5A,B).

## Discussion

Plant chemical defense is often formed as a dual-partner system composed of glycosylated secondary compounds and dedicated detonating β-glucosidases ensuring that a reactive defensive aglycone is released only after deglycosylation (Morant et al., 2008; Piasecka et al., 2015). In Oleaceae species, the oleuropein/OeGLU system serves as a mighty chemical arsenal against herbivores (Konno et al., 1999) and this enzymatic reaction also determines the quality and flavor of olive oil (Romero-Segura et al., 2012; Hachicha Hbaieb et al., 2015; Vitaglione et al., 2015). Previous *in vitro* determination of enzymatic properties of OeGLU (Koudounas et al., 2015, 2017) urged us to functionally characterize this enzyme in the olive tree.

Molecular analyses of the Agroinoculated olive plants confirmed the successful VIGS-mediated silencing of *OeGLU*, and transcript abundance was reduced by more than 80%. VIGS processes, as all the RNA interference (RNAi) approaches, are known to result in a broad range of silencing (Meins et al., 2005), and comparable transcript reduction was observed in the woody tree *Jatropha curcas* using TRV-based constructs (Ye et al., 2009). *In vitro* enzymatic assays with oleuropein revealed that the relative deglycosylation activity of leaf protein extracts from *OeGLU*-silenced plants was drastically reduced compared to control plants and was arrested to the ratio of almost one-third independently of the incubation time of the enzymatic reactions. In contrast, enzymatic assays with pNPGlu confirmed that the relative non-specific β-glucosidase activity of the crude extracts remained at similar levels. In agreement with previously determined biochemical characteristics and kinetics of heterologously expressed OeGLU (Koudounas et al., 2017), these results further confirm that OeGLU is the major oleuropein-specific β-glucosidase in olives since the deglycosylation activity of oleuropein was directly related to the abundance of *OeGLU* transcripts *in planta*. VIGS-mediated silencing is a powerful tool to study the biosynthetic pathway of secoiridoids in the olive tree, and this approach is expected to complement the functional characterization of other enzymatic hubs.

The *in planta* amount of oleuropein was found significantly lower in *OeGLU*-silenced olive plants compared to controls. The content of upstream and downstream secoiridoids was also reduced or even non-detectable revealing that reduction of *OeGLU* transcripts affected unexpectedly this pathway. The characteristic peak at 5.95 ppm that corresponds to H-1 of oleoside 11-methyl ester (Park et al., 1999; Mousouri et al., 2014), the first secoiridoid of the biosynthetic pathway of oleuropein (Figure 1), was non-detectable. Additionally, no differences between control and *OeGLU*-silenced olive plants were observed in the characteristic peaks of precursor iridoids (Figure 1), such as 7-deoxyloganic acid (5.20, 7.41 ppm) (Teng et al., 2005), 7-*epi*-loganic acid (5.38, 7.37 ppm) (Damtoft et al., 1993), 7-*epi*-loganin (5.33, 7.41 ppm) (Itoh et al., 2005), and 7-ketologanin (5.51, 7.39 ppm) (Kamoldinov et al., 2011); therefore, none of these compounds was accumulated in the *OeGLU*-silenced plants.

In contrast, the amount of characteristic triterpenoids was unaffected by *OeGLU* silencing. Although a possible effect in other classes of terpenoids cannot be excluded, the effect was apparently secoiridoid specific. These results strongly suggest that a regulatory loop mechanism controls the flux toward the branch of secoiridoids in olives.

It is worth noting that 7-β-1-D-glucopyranosyl-11-methyl oleoside (Figure 1) has been proposed to be an intermediate in the biosynthetic pathway of olive secoiridoids (Obied et al., 2008) only as the precursor of ligstroside but it is unknown whether it is deglycosylated back to oleoside 11-methyl ester (Damtoft et al., 1993). This compound is found in trace amounts in olive leaves (De Nino et al., 1999) and has not been tested as a potential substrate of OeGLU since it is not commercially available. Even if OeGLU could potentially deglycosylate this diglucoside, the absence of the characteristic peak of its direct precursor – oleoside 11-methyl ester (Figure 1) – is an unexpected result.

In our experimental setup, olive leaves were dried at room temperature for a week prior to extraction and NMR spectra analysis. Although all secoiridoids and secoiridoid glucosides are not volatile compounds, this could potentially have an impact on volatile compounds found at the very early steps of the biosynthetic pathway before iridodial (Figure 1), like for example in the case of 8-hydroxygeraniol. On the other hand, no differences could be observed in the early iridoids among the samples, therefore most likely the biosynthesis of iridoids was supplied with comparable quantities of precursor molecules.

Among the characterized dual-partner defense systems, the absence of one partner typically does not affect the other. For example, cyanogenic glucosides are biosynthesized in barley (*Hordeum vulgare*) leaves despite the lack of co-localized cyanide releasing β-glucosidase and transient overexpression of a cyanogenic β-glucosidase from sorghum (*Sorghum bicolor*) reconstitutes cyanogenesis in barley leaves (Nielsen et al., 2006). In white clover (*Trifolium repens*) either both cyanogenic glucosides and the respective β-glucosidase or one or even none of the two partners are present (Olsen et al., 2007, 2008). In the glucosinolate-myrosinase defense system of Brassicaceae, knockout mutations of two functional myrosinases (TGG1 and TGG2) in *Arabidopsis thaliana* resulted in slightly higher glucosinolate content in certain developmental stages (Barth and Jander, 2006).

One example of unexpected phenotype was recently reported in the medicinal plant *Catharanthus roseus* after unbalancing the metabolic flux of strictosidine biosynthesis – a monoterpene indole alkaloid closely related to oleuropein with similar protein-crosslinking activity after deglycosylation (Guirimand et al., 2010; Sudžuković et al., 2016). VIGS-mediated silencing of either strictosidine β-glucosidase (Carqueijeiro et al., 2021) or the vacuolar exporter of strictosidine (Payne et al., 2017) resulted in necrotic symptoms. Possibly olives have evolved a regulatory framework within the secoiridoid pathway to avoid any substantial accumulation of cytotoxic intermediates that eventually could result in cell death.

A growing body of evidence highlights that the secondary metabolism may regulate plant defense responses which suggests the existence of synergies among distinct biosynthetic pathways (Erb and Kliebenstein, 2020). Mutants of enzymes involved in the biosynthesis of glucosinolates in *Arabidopsis* may affect the pathogen-triggered callose regulation (Clay et al., 2009), the biosynthesis of phenylpropanoids (Kim et al., 2020), hormonal signaling (Burow et al., 2015), or biosynthesis of other tryptophan-derived compounds (Frerigmann et al., 2016). Similar regulatory cross-talks exist in the biosynthesis of benzoxazinoids (Ahmad et al., 2011; Meihls et al., 2013; Li et al., 2018). Even though, feedback or feedforward regulation among specialized metabolism and distinct biosynthetic pathways was observed, in most cases, the underlying molecular etiology remains elusive.

Reports about regulatory loop mechanisms that self-govern a specialized biosynthetic pathway in plants are scarce. Identified examples include epistasis in carotenoid biosynthesis (Kachanovsky et al., 2012) and feedback regulation through intermediates in phenylpropanoids (Blount et al., 2000). To the best of our knowledge, the only known example partially resembling the phenotype observed in *OeGLU*-silenced olive plants has been reported in opium poppy (*Papaver somniferum*). Silencing of codeinone reductase (COR) resulted in accumulation of (*S*)-reticuline, an intermediate compound upstream of seven enzymatic steps proposing a feedback regulatory loop mechanism (Allen et al., 2004) and preventing intermediates of benzylisoquinoline synthesis to enter the morphine-specific branch. In agreement with the absence of any transcriptional effect in other enzymes engaged in the biosynthetic pathway of secoiridoids after silencing *OeGLU* in olives, silencing of *COR* did not affect the abundance of transcripts encoding for enzymes engaged upstream and downstream of (*S*)-reticuline (Allen et al., 2004). Although oleosides- and morphinan- type alkaloids are structurally and biosynthetically different, a fundamental regulatory mechanism could govern both secondary metabolic pathways. The subcellular compartmentalization of the secoiridoid pathway in Oleaceae engaging at least two physically separated compartments at the last enzymatic steps (i.e., vacuole and nucleus) (Koudounas et al., 2017) points out retrograde signaling and a sophisticated cross-talk within the cell.

Biosynthesis of specialized metabolites has energetic and metabolic costs for the plants (Gershenzon, 1994; Neilson et al., 2013; Cipollini et al., 2018). Possibly the absence of OeGLU *in planta* is perceived as a failure in the defensive pathway of secoiridoids and olives completely bypass the flux toward oleuropein to recoup the costs and avoid investing resources in the absence of defensive benefits. The molecular mechanism(s) by which olive cells sense the loss of a key enzymatic hub and bypass the flux toward the biosynthetic pathway of secoiridoids remain to be addressed. Although no difference was observed at the transcript levels of the selected genes engaged in this pathway, possibly the expression levels of other regulatory genes, such as transcription factors or specialized transporters, may have been altered after the silencing of *OeGLU*, and a comparative transcriptomic approach is expected to shed light on the etiology of the observed phenotype. Nonetheless, this study highlights an unexpected direct link between OeGLU and total content of secoiridoids, thus this enzyme could serve as a molecular target of high biotechnological interest in order to produce tailor-made olive oils with adjustable secoiridoid content having an optimized balance of flavor (Vitaglione et al., 2015) and high nutritional value with beneficial aspects in human health.

## Data Availability Statement

The original contributions presented in the study are included in the article/Supplementary Material, further inquiries can be directed to the corresponding author.

## Author Contributions

KK and PH designed the research. KK, MT, and EA prepared the constructs, performed VIGS experiments, analyzed gene expressions, and performed *in vitro* enzymatic assays. EM and PM performed method development for qualitative and quantitative analyses of olive leaf ingredients by NMR. AR analyzed application of NMR methodology and performed data collection. KK, EM, PM, and PH wrote the manuscript. All authors contributed to the article and approved the submitted version.

## Conflict of Interest

The authors declare that the research was conducted in the absence of any commercial or financial relationships that could be construed as a potential conflict of interest.

## Publisher’s Note

All claims expressed in this article are solely those of the authors and do not necessarily represent those of their affiliated organizations, or those of the publisher, the editors and the reviewers. Any product that may be evaluated in this article, or claim that may be made by its manufacturer, is not guaranteed or endorsed by the publisher.

## Footnotes

1
www.arabidopsis.org
