# Supplementary material for: Silencing of Oleuropein β-Glucosidase Abolishes the Biosynthetic Capacity of Secoiridoids in Olives
Source: Front Plant Sci. 2021 Sep 3;12:671487. doi: 10.3389/fpls.2021.671487 (PMC8446429; doi:10.3389/fpls.2021.671487)
Supplement: Supplementary file 1 [file Data_Sheet_1.PDF]

Supplementary Material

Silencing of oleuropein *β*-glucosidase abolishes the biosynthetic capacity of secoiridoids in olives.

Konstantinos Koudounas[1,3,4], Margarita Thomopoulou[1,4], Aimilia Rigakou[2], Elisavet Angeli[1], Eleni Melliou[2], Prokopios Magiatis[2], Polydefkis Hatzopoulos[1]*

[1]Laboratory of Molecular Biology, Department of Biotechnology, Agricultural University of Athens, 11855 Athens, Greece

[2]Laboratory of Pharmacognosy and Natural Products Chemistry, Department of Pharmacy, National and Kapodistrian University of Athens, 15771 Athens, Greece

[3]Present address: EA2106 Biomolécules et Biotechnologies Végétales, Université de Tours, 37200 Tours, France

[4]These authors contributed equally to this work

* To whom correspondence should be addressed: phat@aua.gr

This file includes:

- Supplementary Figures 1 to 7
- Supplementary Tables 1 and 2
- Supplementary References

a

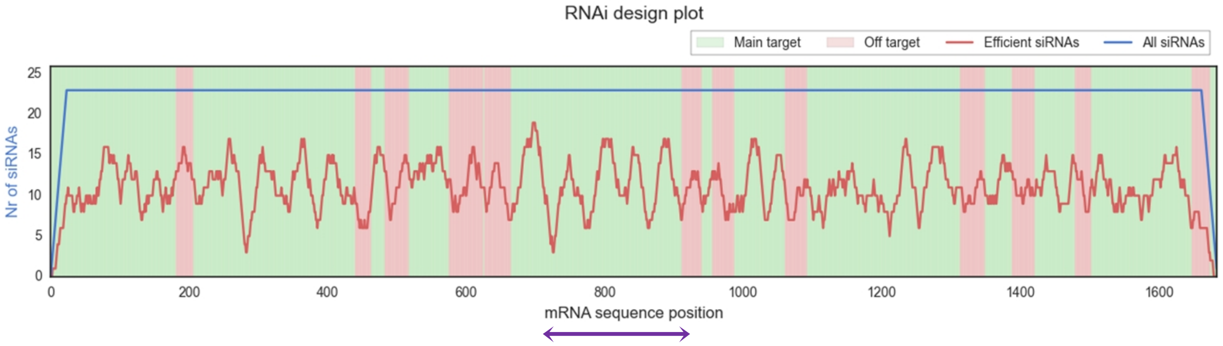

b

Targets	Total siRNA hits	Efficient siRNA hits
Oeu028845.1	189	96

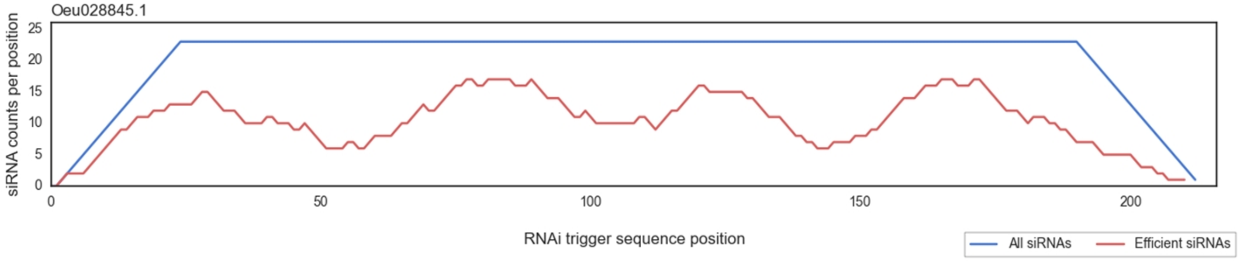

Supplementary Figure 1. Bioinformatic analysis for the prediction of specific regions that do not trigger off-target silencing. (**a**) RNAi design plot using the corresponding coding sequence (CDS) of OeGLU from the recently released genome (Unver et al., 2017) of olive (Oeu028845.1) against the 60,214 predicted CDSs. (**b**) In the selected region to be cloned (indicated as a double arrowed purple line corresponding to region 695-905 nts of the CDS of OeGLU; AY083162.1), no siRNA of 23nts length was predicted to trigger off-target silencing. Analysis was performed with si-Fi (Lück et al., 2019).

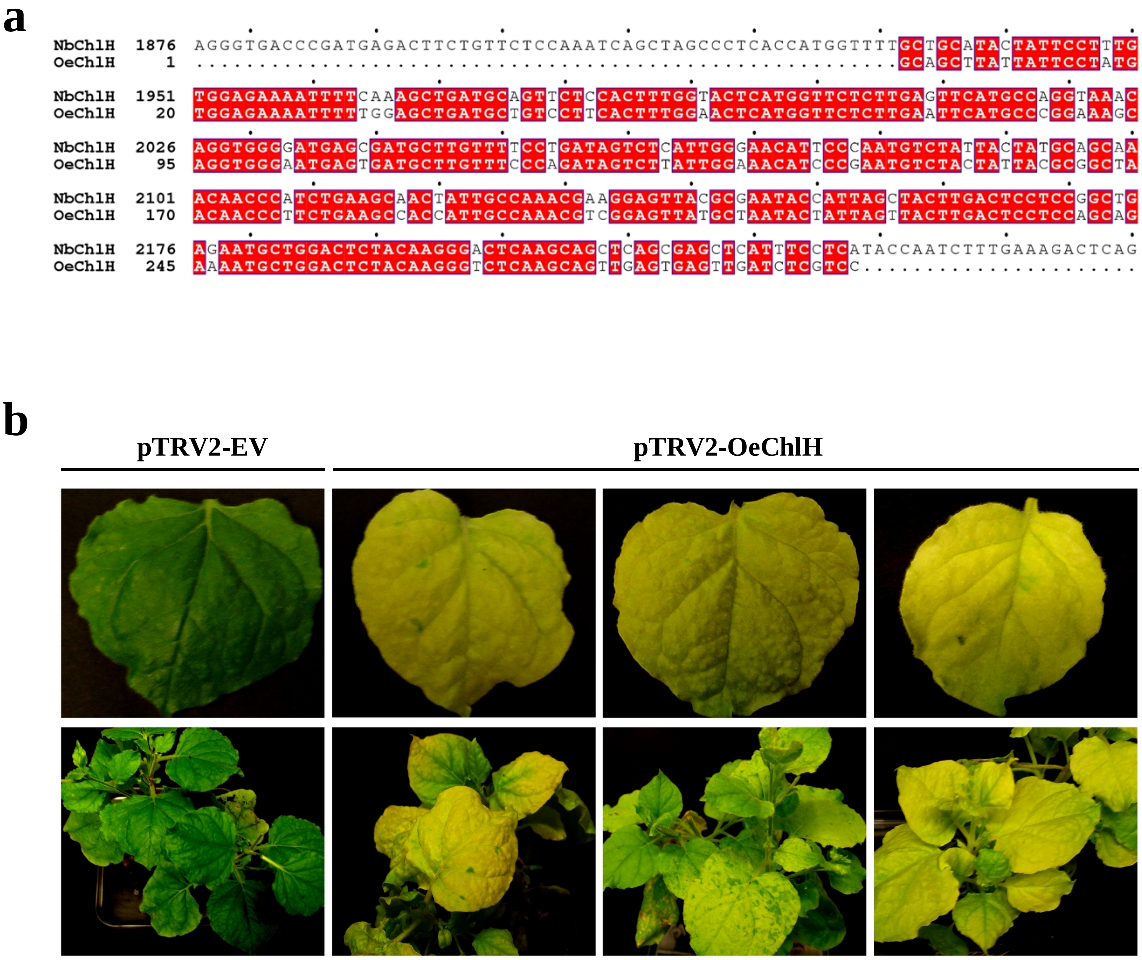

Supplementary Figure 2. (**a**) Alignment of tobacco *NbChlH* (GenBank Acc. no.: KA736544.1) with the corresponding fragment of *OeChlH* from olive (GenBank Acc. no.: GABQ01080755.1). Alignment was performed using ClustalX 1.83 software (http://www.clustal.org) and graphically displayed with the ESPript 3.0 (Robert and Gouet, 2014) server (http://espript.ibcp.fr). (**b**) *N. benthamiana* plants Agroinfiltrated with pTRV1 and either pTRV2-EV (empty vector) or pTRV2-OeChlH constructs.

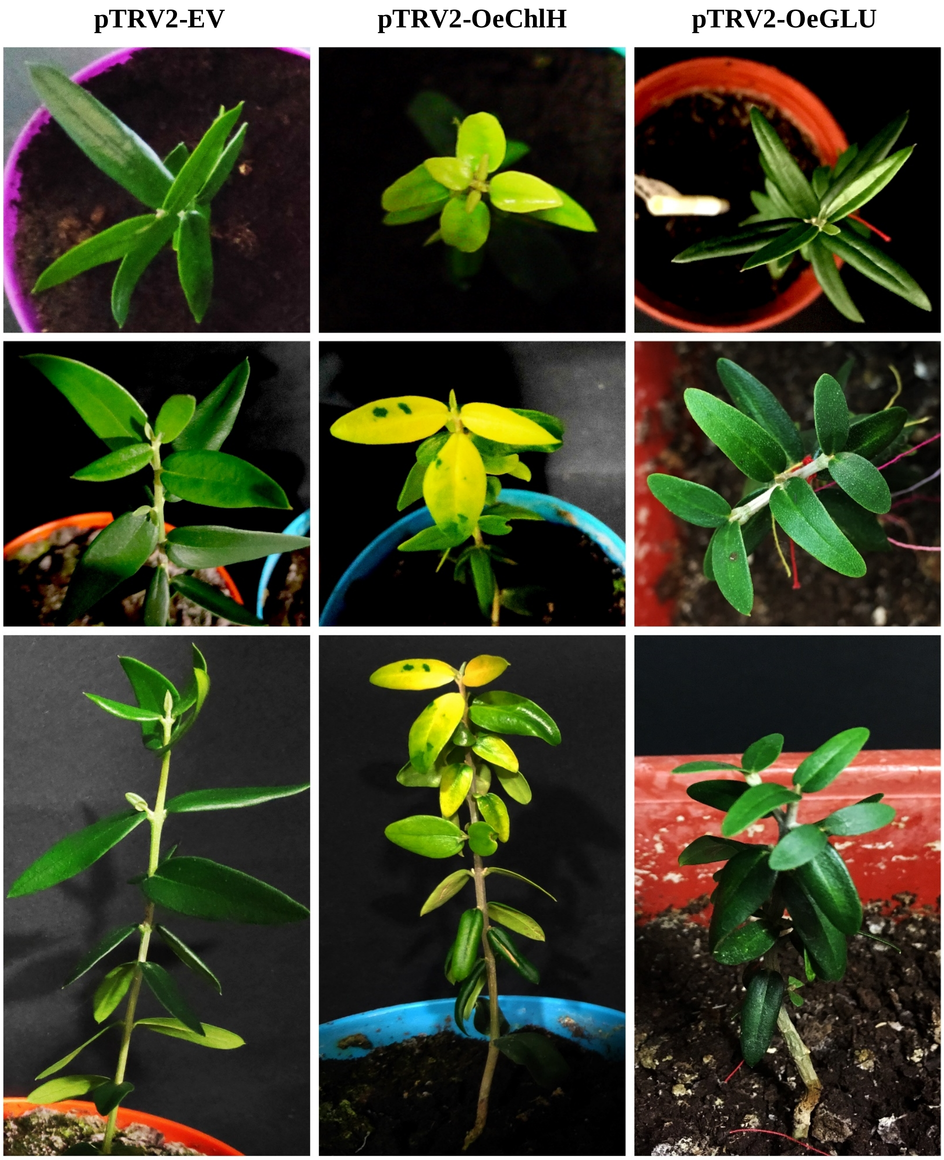

Supplementary Figure 3. Representative phenotypes observed in olive plantlets Agroinoculated with pTRV1 and either pTRV2-EV or pTRV2-ChlH or pTRV2-OeGLU constructs.

a

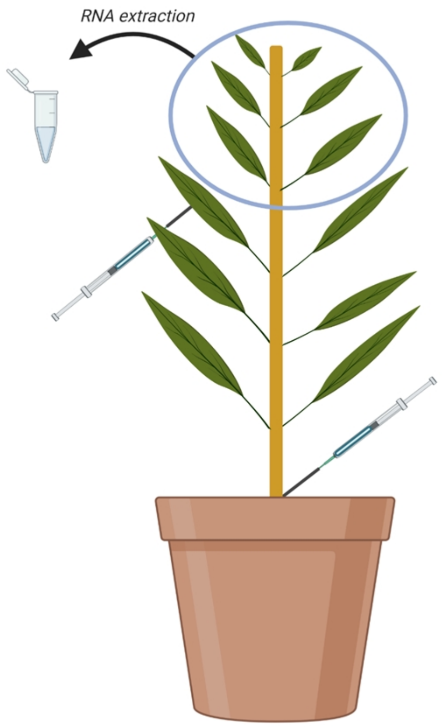

b

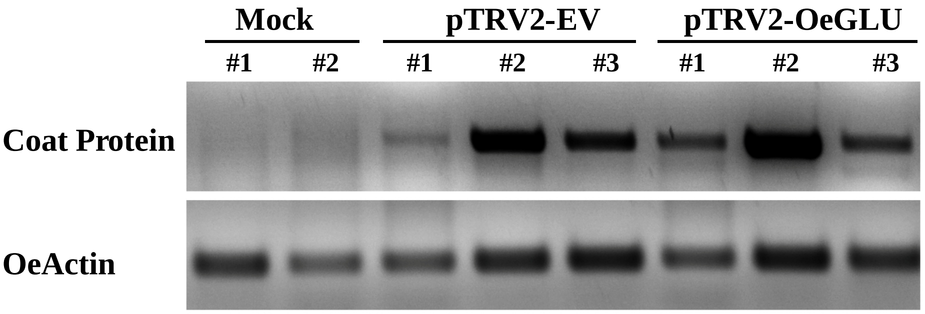

Supplementary Figure 4. (**a**) Schematic representation of VIGS Agroinoculation in olive. Agrobacteria harboring either the pTRV2-EV or the pTRV2-OeGLU constructs were mixed (1:1) with Agrobacteria harboring the pTRV1 construct and were co-inoculated onto the abaxial side of the leaves and on the crown region by a syringe. RNA was extracted from newly emerged (i.e. non-Agroinoculated) leaves for further analyses. Created with Biorender.com. (**b**) Detection of transcripts encoding for TRV Coat Protein (GenBank Acc. no.: AF406991.1) in newly emerged leaves of untreated plants (Mock) or plants Agroinoculated with either pTRV2-EV or pTRV2-OeGLU constructs. Numbers indicate biological replicates.

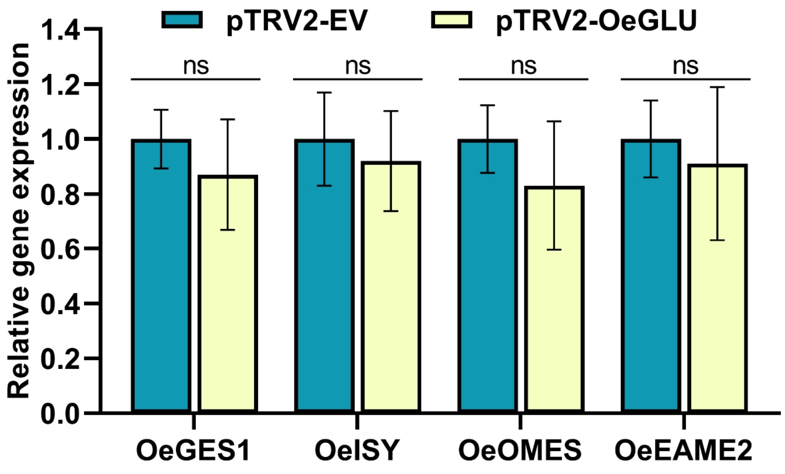

Supplementary Figure 5. Relative expression levels of characterized genes engaged in the secoiridoid pathway (Figure 1). Three technical replicates of three biological replicates per treatment were analyzed. OeGES1, geraniol synthase; OeISY, iridoid synthase; OeOMES, oleoside methyl ester synthase; OeEAME2, elenolic acid methylesterase 2. Amplification of OeSXS (secoxyloganin synthase) and OeEAME1 (elenolic acid methylesterase 1) transcripts could not be achieved in the analyzed tissue (i.e. young leaves of cv. “Koroneiki”). Mean ± SE (ns: not significant; Student’s *t*-test).

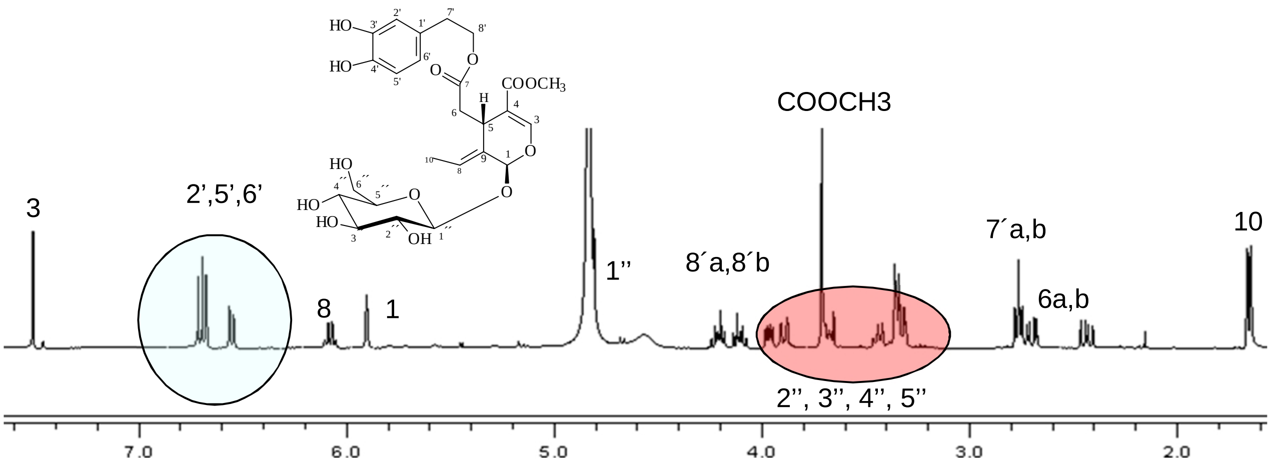

Supplementary Figure 6. ^{1}H-NMR spectrum of pure oleuropein in CD_3OD.

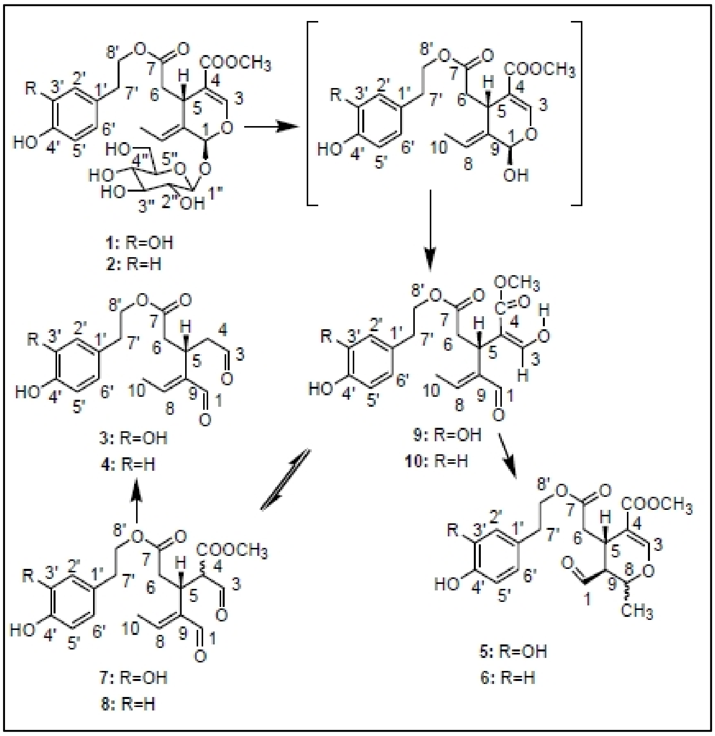

Supplementary Figure 7. Proposed biosynthetic pathway explaining the transformation of the precursor molecules oleuropein (**1**) and ligstroside (**2**) to the aglycon forms (**5**, **6**, **7**, **8**, **9**, **10**) and the decarboxymethyl derivatives oleocanthal (**4**) and oleacein (**3**).

Primer	Sequence(5’-3’)
**oligo(dT)$_{17}$**	GTCGACCTCGAGTTTTTTTTTTTTTTTTT
**OeChlHi-F**<br>**OeChlHi-R**	GCAGCTTATTATTCCTATGTGG<br>GGACGAGATCAACTCACTCAAC
**OeGLUi-F**<br>**OeGLUi-R**	AGCATGCCCGTCTTCATAGAG<br>TGAGAGTCAGTAAGAGGCTCA
**OeActin_q-F**<br>**OeActin_q-R**	GTATGTTGCTATCCAGGCTGTT<br>AAATGGGTACTGTGTGACTCAC
**OeGLUq-F**<br>**OeGLUq-R**	GCTGCTGGATTCAGTGTTAGA<br>GGTGCTGCCTCTAAGCCTTTT
**TRV2cp-F**<br>**TRV2cp-R**	CTGGGTTACTAGCGGCACTGAATA<br>TCCACCAAACTTAATCCCGAATAC
**OeGES1q-F**<br>**OeGES1q-R**	AACCATATCCGAAACTCTTTTCCGC<br>CACACAATCTATGTTCTTCTCTCT
**OeISYq-F**<br>**OeISYq-R**	AAGGATAAGGACTCCGTGTGG<br>GCTCAGCACATTCTCAAGACAA
**OeOMESq-F**<br>**OeOMESq-R**	CCAACTCCAAGGATGGGTCC<br>TGGCTTGTTTGGTCAATTCAGAC
**OeSXSq-F**<br>**OeSXSq-R**	CGGGTGAAATACGGCAAATTATTTC<br>CCTTCATAAGTCCAAAGATTTCTTCG
**OeEAME1q-F**<br>**OeEAME1q-R**	TCTTGGATGGACACTCAATTCG<br>TTGTGGCCGGCAAGTATTTCGG
**OeEAME2q-F**<br>**OeEAME2q-R**	GAGGCCAGGCTCTTTTTTTATGG<br>GTGATCAGTATTTTCCGCTTCC

Supplementary Table 1. List of primers used in this study.

	Oleuropein (mg)		
**Repetition**	pTRV2-EV	pTRV2-OeGLU	pTRV2-OeGLU
**1**	3.80	2.42	ND
**2**	7.89	4.13	1.27
**3**	6.14	0.82	ND

Supplementary Table 2. Oleuropein content (mg) per 100 mg (dry weight) of olive leaves. Olive plantlets Agroinoculated either with pTRV2-EV (n=3) or with pTRV2-OeGLU (n=6) constructs were screened. ND: non detected.

Supplementary References

Lück, S., Kreszies, T., Strickert, M., Schweizer, P., Kuhlmann, M., and Douchkov, D. (2019). siRNA-Finder (si-Fi) software for RNAi-target design and off-target prediction. *Frontiers in Plant Science* 10. doi: 10.3389/fpls.2019.01023

Robert, X., and Gouet, P. (2014). Deciphering key features in protein structures with the new ENDscript server. *Nucleic Acids Research* 42**,** W320-W324. doi: 10.1093/nar/gku316

Unver, T., Wu, Z., Sterck, L., Turktas, M., Lohaus, R., Li, Z., et al. (2017). Genome of wild olive and the evolution of oil biosynthesis. *Proceedings of the National Academy of Sciences* 114**,** E9413-E9422. doi: 10.1073/pnas.1708621114
